# The first case of benign multicystic mesothelioma presenting as a splenic mass

**DOI:** 10.3332/ecancer.2016.678

**Published:** 2016-10-04

**Authors:** Antonio D’Antonio, Carlo Baldi, Maria Addesso, Carmine Napolitano

**Affiliations:** 1 Department of Pathologic Anatomy, AOU S Giovanni di Dio e Ruggi D’Aragona, via S Leonardo, Salerno 84100, Italy; 2 Unit of Pathologic Anatomy, Hospital Scarlato ASL SA, Pagani (SA) 84013, Italy; 3 Unit of Surgery, AOU S Giovanni di Dio e Ruggi D’Aragona, via S Leonardo, Salerno 84100, Italy

**Keywords:** multicystic mesothelioma, spleen, peritoneum, immunohistochemistry, prognosis

## Abstract

Multicystic mesothelioma (MM) is a relatively rare tumour arising in the pelvic peritoneum of the tuboovarian region of young woman. Exceptionally, MM occurs on the serosal surfaces of various organs including kidney, bladder, lymph nodes, and liver. We report here the first case of MM wherein a 58-year-old woman with a previous history of endometriosis of the right ovary presented with a large multicystic mass of the spleen. The diagnosis of MM was made on a surgical specimen after splenectomy. A histopathologic examination is always necessary for the diagnosis of MM which should be differentiated from other lesions particularly from cystic lymphangioma. At one year follow-up, the patient had no evidence of recurrence. Despite the high frequency of local recurrences, MM is a benign lesion and ‘en bloc’ surgical excision with prolonged follow-ups is the treatment of choice.

## Background

Multicystic mesothelioma (MM), is a relatively rare tumour which occurs mainly in young women in their reproductive age [1–29]. MM arises in most instances from the mesothelial lining of the pelvic peritoneum, usually in the tuboovarian region. Secondary involvement of the serosa of other organs has been reported [12–14, 23]. This tumour has been given different names including benign cystic mesothelioma [5,18–20], multilocular peritoneal inclusion cyst [6, 15], postoperative peritoneal cyst [16], inflammatory inclusion cyst of peritoneum [17], and others [22, 23]. All these different terms reflect the unclear origin of the disease. The current consensus is that MM is not a true neoplasm, but a reactive proliferation because of trauma, surgery, or chronic inflammation [13, 15]. Up to date approximately 150 cases have been reported all over the world. We would like to report here the first case of MM originating in the spleen of the female patient with a previous diagnosis of endometriosis of the right ovary treated with splenectomy.

## Clinical case

A 58-year-old woman with a surgical history of right oophorectomy for ovarian endometriosis presented with weakness and left flank pain with bulging of the left side of the abdomen. The tumour marker test showed normal findings (α-fetoprotein 0,9 ng/mL, carcinoembryonic antigen 2,1 ng/mL), except for an elevated serum carbohydrate antigen of 125 (>625 U/mL). Contrast enhanced computed tomography (CT) of abdomen and pelvis showed a large multicystic mass of the spleen. A vascular proliferation of the spleen was clinically suspected and a surgical splenectomy was planned. A vascular proliferation of the spleen was clinically suspected and a splenectomy was carried out. Patient had an uneventful postoperative period and was discharged in postoperative day (POD) seven. Around three and six months after surgery, the patient had no complaints and ultrasonography of abdomen was normal. At one year after surgery, a CT scan showed no disease recurrence.

## Materials and methods

The tissues were fixed in 4% buffered formalin, routinely processed, and embedded in paraffin. After which 3–4 *μ*m-thick sections were stained with haematoxylin and eosin, Immunohistochemistry was conducted with a Benchmark automated staining system (Ventana Medical Systems, Tucson, AZ) using Ventana monoclonal antibodies (calretinin 1:2000; mesothelial Ag HMBE-1 1:50; CD31 1.100; CD34 1.50; FVIII 1:200; smooth muscle actin [SMA] 1:40; pancytokeratin [CKpan] 1:100; CK5/6 1:100; oestrogen receptor [ER] 1:100). Appropriate positive and negative controls were used.

## Results

**Figure 1.**
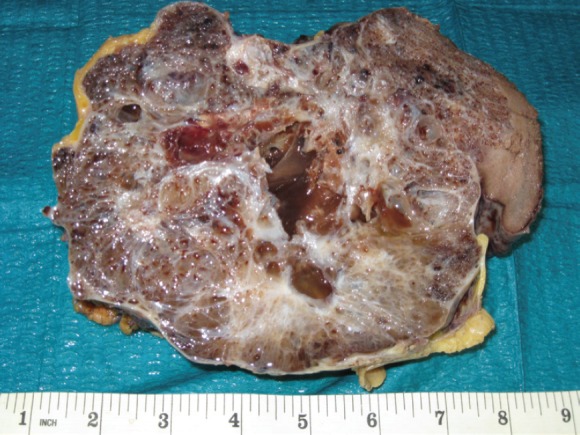
The cut surface of the spleen showed a large mass with numerous macrocystic and microcystic spaces filled by gelatinous or haemorrhagic fluid.

The spleen measured 20 x 10 cm and the cut sections showed a large mass with numerous macrocystic and microcystic spaces filled by gelatinous or haemorrhagic fluid (Figure 1). Histologically the macrocystic and microcystic spaces were lined by flattened endothelial-like cells or cuboidal cells with bland round nuclei (Figure 2a). Some areas showed a lymphangioma-like pattern for the presence of cystic spaces contained erythrocytes (Figure 2b). Tubular and gland-like spaces with clear epithelioid lining cells were also present (Figure 2c). Atypia and mitoses were absent. A cuboidal epithelium with squamous metaplasia lined some cystic nodules filled with eosinophilic proteinaceous material (Figure 2d). In all cases immunohistochemistry revealed diffuse positivity of the lining epithelium for CKPan, calretinin (Figure 3a), HMBE-1, CK5/6, and ER (Figure 3b) with negativity for CD 34, CD31, FVIII.

## Discussion

Multicystic mesothelioma (MM) is a relatively rare but well-known clinicopathologic entity, first described in 1979 by Menemeyer and Smith [1]. To date approximately 150 cases have been reported in the literature [1–29]. Typically MM arises from the pelvic peritoneum around the tuboovarian region, and it occurs more frequently in young women [2, 13, 15, 18]. A localisation on the serosal surfaces of the pelvic viscera or peritoneum of various organs including appendix [12], lymph nodes, liver [13], and kidney [14]. Although MM occurs most frequently in women in their reproductive age, people of any age and sex can be affected. Clinically MM is asymptomatic and only in presence of large masses of disseminated forms may be associated with pain and ascites [18]. Macroscopically surgical specimen is represented by uniloculated or multiloculated thin-walled cystic nodules ranging few millimeters to several centimeters filled of clear, gelatinous, or haemorrhagic fluid [2–13]. Occasionally, the cystic wall contained solid mural nodules [15]. Histologically the cystic spaces are separated by a fibrous, edematous, or myxoid stroma, with a rich vascular network. Tubular-like or gland-like spaces can be present. These spaces are lined by an endothelial-like epithelium or cuboidal epithelioid-like cells with clear cytoplasm [6, 13]; squamous metaplasia is present in some cases [6]. A differential diagnosis should also include cystic lymphangioma which generally spares the pelvis and histologically shows bundles of smooth muscle and lymphoid aggregates in the cystic wall, absent in the MM. Further in the spleen because of the presence of a multicystic lesion, a vascular proliferation (haemangioma, haemangiomatosis, angiosarcoma) should always be excluded [29]. In some cases MM may show a haemangioma-like or lymphagioma-like pattern with cystic spaces containing haemorrhagic fluid and haemosiderin in the stroma. Immunohistochemistry helps in the differential diagnosis. MM is positive for calretinin and negative for endothelial markers as CD31, CD34, and F-VIII. The absence of atypia and few or absent mitosis are the diagnostic clue to differentiated MM from malignant mesothelioma and other malignant epithelial neoplasms (for example in cases of MM characterised by tubular spaces with clear cells). Though these should be done in association with immunohistochemistry. The pathogenesis of MM is controversial and remains unclear. Some authors believe that MM is a true tumour because they have high tendency to recur locally after surgical resection. The literature, however, tells that only two cases have been reported of malignant transformation, [30–31] and only one death was reported because of this lesion [32]. The majority of cases have an excellent prognosis and probably the presence of local recurrences is because of the number of cystic lesions and extension of disease that makes surgical resection very difficult. We, as other authors, favour a reactive nature of MM because of various factors that may promote the proliferation of mesothelium with formation of multiple uni or multilocular cysts. These then go on to form large pelvic or intra-abdominal masses or visceral nodules. In fact most patients presented has had previous history of either abdominal surgery, pelvic inflammation, trauma [6, 13, 15–17], or an association with endometriosis [33]. The therapy of MM is surgery. The complete excision of cystic masses if possible may prevent recurrences [6, 11, 34, 35] Recurrences occur more frequently in large masses [36] or disseminated disease and treated by hormonal therapy [37], hyperthermic intraperitoneal chemotherapy using cisplatin or doxorubicin [38], and sclerotherapy [1–10, 39]. In literature, there have been some cases reported of MM positivity for oestrogen receptor [40]. The influence of oestrogen receptor on MM pathogenesis and cyst growth is not well understood. Although immunohistochemical detection of sex hormone receptors in MM is uncommon [40], the focal presence of ER and/or PR in these cases have been used by some authors as support for a therapy with gonadotropin-releasing hormone agonist and the anti-oestrogen agent [41–42]. The biological rationale for this response, however, remains unexplained.

## Conclusions

Multicystic mesothelioma is a benign and reactive lesion that can mimic many different disease entities including ovarian malignancies and cystic lymphangioma on imaging, and therefore histopathologic examination is always necessary for the diagnosis. Surgery is reported to be the only effective treatment for MM with a high risk of local recurrence if not completely excised. Further surgery, prolonged follow up of these pateints is necessary.

## Conflicts of interest

The authors declare that they do not have any financial or potential conflicts of interest of any kind.

## Authors’ contributions

AD proposed the study. CB and MA performed the research and wrote the first draft. CN collected and analysed the data. All authors contributed to the design and interpretation of the study and to further drafts. AD is the guarantor.

## Funding

None.

## Ethical approval

Not needed.

## Figures and Tables

**Figure 2a.** The cystic spaces were lined by flattened endothelial-like cells or cuboidal cells with bland round nuclei. (HE 20x).

**Figure 2b.** A lymphangioma-like pattern was also evident (HE 10x).

**Figure 2c.** Note tubular and gland-like spaces with clear epithelioid lining cells (HE 40x).

**Figure 2d.** Some cyst showed squamous metaplasia (HE 20x).

**Figure 3a.** Strong immunorectivity of the lining cells with calretinin (immunoperoxydase 20x).

**Figure 3b.** Some cyst showed positive nuclei for ER (immunoperoxydase 20x).
